# Correction: The increasing incidence of visceral leishmaniasis relapse in South Sudan: A retrospective analysis of field patient data from 2001–2018

**DOI:** 10.1371/journal.pntd.0011952

**Published:** 2024-02-12

**Authors:** Gabriel Naylor-Leyland, Simon M. Collin, Francis Gatluak, Margriet den Boer, Fabiana Alves, Abdul Wasay Mullahzada, Koert Ritmeijer

There are errors in the Funding and Competing Interests statements. The correct statements are:

**Funding:** MSF provided support for this study in the form of salaries for FG, MdB, AWM, and KR. The specific roles of these authors are articulated in the ‘author contributions’ section. MSF otherwise had no role in study design, data collection and analysis, decision to publish, or preparation of the manuscript.

**Competing Interests**: The authors have read the journal’s policy and have the following competing interests to declare: MSF provided support for this study in the form of salaries for FG, MdB, AWM, and KR. This does not alter our adherence to *PLOS Neglected Tropical Diseases* policies on sharing data and materials. There are no patents, products in development or marketed products associated with this research to declare.
