# Supplementary material for: The increasing incidence of visceral leishmaniasis relapse in South Sudan: A retrospective analysis of field patient data from 2001–2018
Source: PLoS Negl Trop Dis. 2022 Aug 18;16(8):e0010696. doi: 10.1371/journal.pntd.0010696 (PMC9426874; doi:10.1371/journal.pntd.0010696)
Supplement: S1 Table — (PDF) [file pntd.0010696.s001.pdf]

Table S1: Médecins Sans Frontières (MSF) visceral leishmaniasis (VL) programme data from Lankien and patient-level data from all MSF sites in South Sudan, 2001-2018

	**Programme data (Lankien)**		**Patient-level data (all sites)**	
**Year**	**Primary VL (%)**	**VL relapse (%)**	**Primary VL (%)**	**VL relapse (%)**
2001	615 (97.0%)	19 (3.0%)	1637 (95.9%)	70 (4.1%)
2002	1420 (97.9%)	31 (2.1%)	2653 (93.6%)	183 (6.5%)
2003	2207 (92.3%)	184 (7.7%)	2933 (92.9%)	223 (7.1%)
2004	422 (83.7%)	82 (16.3%)	483 (95.3%)	24 (4.7%)
2005	746 (95.2%)	38 (4.9%)	6 (75.0%)	2 (25.0%)
2006	356 (81.3%)	82 (18.7%)	62 (65.3%)	33 (34.7%)
2007	127 (88.2%)	17 (11.8%)	no data	no data
2008	53 (86.9%)	8 (13.1%)	no data	no data
2009	172 (96.1%)	7 (3.9%)	168 (99.4%)	1 (0.6%)
2010	964 (95.5%)	46 (4.6%)	847 (94.2%)	52 (5.8%)
2011	437 (88.6%)	56 (11.4%)	120 (89.6%)	14 (10.5%)
2012	194 (89.0%)	24 (11.0%)	170 (90.0%)	19 (10.0%)
2013	1288 (96.3%)	50 (3.7%)	1360 (99.1%)	12 (0.9%)
2014	4202 (91.6%)	387 (8.4%)	4158 (91.2%)	399 (8.8%)
2015	1004 (71.8%)	395 (28.2%)	985 (70.6%)	410 (29.4%)
2016	1299 (91.2%)	126 (8.8%)	1285 (90.1%)	142 (10.0%)
2017	1567 (87.7%)	219 (12.3%)	1564 (87.3%)	228 (12.7%)
2018	742 (74.0%)	261 (26.0%)	741 (74.0%)	261 (26.0%)
