# Supplementary material for: The increasing incidence of visceral leishmaniasis relapse in South Sudan: A retrospective analysis of field patient data from 2001–2018
Source: PLoS Negl Trop Dis. 2022 Aug 18;16(8):e0010696. doi: 10.1371/journal.pntd.0010696 (PMC9426874; doi:10.1371/journal.pntd.0010696)
Supplement: Attachment — Submitted filename: Responses to Reviewers Comments SM.docx [file pntd.0010696.s004.docx]

Responses to Reviewers’ Comments

Reviewer #1

1. What is the definition of relapse /Re-infection?

**We have added a sentence to our Methods (under ‘Patient-level data’): “*A relapse case was defined as any patient who was recorded with a diagnosis of ‘Relapse VL’ on admission, based on the attending medical staff asking the patient whether they had previously been treated for VL more than 30 days before admission for the current VL episode.*” We have added a sentence to our Discussion (under ‘Limitations’) explaining that “*****Differentiation between relapse and reinfection can only be established by isoenzyme characterisation, which is not possible in routine programme settings. However, very limited research (in VL patients with HIV co-infection) indicates that cases presenting with a history of previous VL episodes are much more likely to be relapse than re-infection.”* citing Morales *et al.* JID 2002*.***

2. How were the cases diagnosed?

**Diagnosis in relapse patients was via tissue aspiration (mostly splenic) and microscopy. We have added this detail to our Methods.**

3. What are the side effects of the drugs being used for e.g., SAG , PM,LAMB ?

**An overview of the side effects of SSG/PM combination therapy is given in the following reference: Kimutai *et al.* Safety and Effectiveness of Sodium Stibogluconate and Paromomycin Combination for the Treatment of Visceral Leishmaniasis in Eastern Africa: Results from a Pharmacovigilance Programme. Clin Drug Investig 2017, 37(3):259-272. Side effects of L-AMB in doses similar to those used for Eastern Africa (30 mg/kg total dose) are mild. See below reference:**

**Bern C, Adler-Moore J, Berenguer J, Boelaert M, den Boer M, Davidson RN, Figueras C, Gradoni L, Kafetzis DA, Ritmeijer K, Rosenthal E, Royce C, Russo R, Sundar S, Alvar J. Liposomal amphotericin B for the treatment of visceral leishmaniasis. Clin Infect Dis. 2006 Oct 1;43(7):917-24.**

4. Is 30 mg/kg recommended for LAMB in VL?

**Yes. This dose is recommended by WHO. VL in Eastern Africa responds less well to AmBisome than in India. We have added to our Methods citations of:**

**Balasegaram, Ritmeijer, Lima *et al.* Liposomal amphotericin B as a treatment for human leishmaniasis. Expert Opin. Emerging Drugs 2012 Dec;17(4):493-510.**

**World Health Organization: Control of the leishmaniases: WHO TRS N°949. In: Report of a meeting of the WHO Expert Committee on the Control of Leishmaniases, Geneva, 22–26 March 2010. Geneva: World Health Organization; 2010**

5. What were the factors associated with relapse?

**It was not the aim of our analysis to identify risk factors for relapse, because most of the VL relapse patients in our dataset could not be matched to their primary VL treatment record. Odds ratios for VL relapse by TB and HIV status could be estimated because these risk factors were recorded in the treatment record for the relapse episode. However, we cite an earlier study from South Sudan (Gorski *et al.* ref. #19) that identified risk factors which would be applicable to this patient population.**

6. What was the treatment of HIV-VL and VL-TB?

**Patients with HIV-VL co-infection received AmBisome. VL-TB patients typically have other symptoms qualifying as severe VL and are therefore often treated with AmBisome. We have added this detail to the text (Methods).**

7. What were drug interactions observed?

**Between SSG and paromomycin there are no relevant interactions. SSG is metabolized in the liver and PM is cleared via the kidneys. There are no known drug interactions between anti-leishmanial drugs and first line TB drugs or first line antiretroviral drugs.**

8. What was the follow up period?

**Patients were not actively followed up but were advised to come back to the clinic in case symptoms re-occurred. We have added this detail to the text (Methods).**

9. What was the rescue therapy?

**In case a patient does not respond to first-line therapy with SSG+PM, we switch to AmBisome. We have added this detail to the text (Methods).**

10. How many cases converted to PKDL and after what length of time?

**It was not our aim to include PKDL as an outcome in our analysis. We note in our Discussion that “*Given the limitations of the current retrospective study, MSF is planning a prospective observational study to assess the incidence of relapse (and PKDL) after treatment for primary VL in South Sudan in which patients will be followed up for 12 months*.” There is only passive case finding for PKDL and most PKDL cases are mild and self-healing within weeks/months. These cases will typically not present. Only patients with severe PKDL present at the hospital for treatment.**

11. Is any elimination program currently running in Sudan?

**There is currently no VL elimination program in any Eastern African country.** **Current national strategies are focused on disease control.**

Reviewer #2

1. Abstract (Conclusion): re. “Increasing incidence of VL relapse in South Sudan does not appear to be explained by changes in patient characteristics or artefact.” Meaning is not clear from the word artefact; more clear word can be used here.

**We have changed ‘artefact’ to ‘other factors’.**

2. Page 3: “Conflict-related challenges to VL control, including lack of infrastructure and clinical staff, internally-displaced populations, and malnutrition [2], have been compounded recently by cuts in aid and COVID-related and longer-term threats to supplies of diagnostic kits and drugs [3, 4].” Year and an estimated % of cuts in aid can be mentioned here.

**The reference to cuts in aid referred specifically to UK overseas aid. We have clarified this by altering the sentence to read “*compounded recently by the complete withdrawal of UK aid funds for VL control in 2020, the COVID-19 pandemic, and longer-term threats to supplies of diagnostic kits and drugs*.”**

3. Page 4: “Drug resistance presents another potential challenge to VL treatment and control [5].” Name of drugs and extent of resistance can be mentioned here, with available references.

**We have re-ordered this and the subsequent paragraph to provide the missing information. It now reads “*Pentavalent antimonials have been the mainstay of treatment against VL for many decades, and resistance to these, as already established on the Indian subcontinent by the early 1990s [5], and potentially to newer therapeutic agents such as miltefosine and paromomycin (PM) [6], presents another potential challenge to VL treatment and control*.” where reference #5 is Sundar, Drug resistance in Indian visceral leishmaniasis. *Trop Med Int Health* 2001, 6(11):849-854.**

4. Page 4: “replaced by a 17-day combination regimen of SSG and paromomycin (PM)” Injectable

**We have inserted the word ‘*injectable’*.**

5. Page 4: “Non-inferiority of SSG/PM compared to SSG monotherapy was proven in clinical trials in east Africa” Better word like higher efficacy can be used here

**We have changed ‘Non-inferiority’ to ‘Equivalent efficacy’. The cited trial was not a non-inferiority trial. It concluded that “*17 day SSG & PM combination treatment had a good safety profile and was similar in efficacy to the standard 30 day SSG treatment*”**

6. Page 4: “Liposomal amphotericin B (AmBisome) was introduced in the late 90’s [11], but its prohibitively high price limited its use to severely ill patients.” Severity can be defined here, what is meant by severely ill patient.

**We have added parenthetically the definition “(*****patients unable to walk or sit up, and with severe weight loss, severe anaemia, severe super-infections, and other markers of poor prognosis)*”.**

7. Page 4: “AmBisome has been provided as a donation by the manufacturer via WHO for these indications since 2012.” Gilead Sciences, Inc.

**We have added ‘Gilead Sciences Inc.’ at this first mention of AmBisome.**

8. Page 4: “In settings such as South Sudan where active patient follow-up is extremely difficult…” Is there any data available for the % of follow up done in treated VL cases? If available, it can be mentioned here with reference.

**We do not have data on % follow-up because it is not routinely performed. We have added this clarification to the text, namely “*In settings such as South Sudan where active patient follow-up is extremely difficult and not routinely performed*.”**

9. Page 5: “The facility provides the only hospital care in a large region,” It can be re-framed for more clarity

**We have rearranged the Methods section on ‘*Study design, setting and population’* to address this and the next two comments.**

10. Page 5: “Due to the dynamic epidemiology of VL in South Sudan, characterised by incidence fluctuations between locations and from year to year…” It can be re-framed for more clarity. Somewhere, operational definition of incidence can be defined.

**We have clarified “*Due to the dynamic epidemiology of VL in South Sudan, characterised by large fluctuations in case numbers between locations and from year to year, with focal and seasonal outbreaks.*”**

11. Page 5: “MSF has a flexible operational model that includes provision of treatment at more than 25 other sites across South Sudan…” Above para has mention that MSF has only one treatment centre in Lankien, Jonglei State, “only hospital care in a large region”. Please reframe the above sentence.

**We have rearranged the Methods section on ‘*Study design, setting and population’* to address this and the preceding two comments and we have clarified that “[the operational model] has included provision of treatment at more than 25 other sites” (not currently at 25 sites).**

12. Page 5: “*Diagnosis and treatment of VL in South Sudan*” An algorithm would be very helpful for easy understanding of diagnosis and treatment protocols.

**We cite *Guidelines for diagnosis, treatment and prevention of visceral leishmaniasis in South Sudan*, which are freely available for interested readers to download and which include a diagnostic algorithm flowchart (Figure 4). We did not consider it necessary to reproduce this figure in our manuscript.**

13. Page 6: “Routine testing of all patients for HIV commenced in 2012” Any available data on % of HIV-VL testing can be mentioned here, like of 95% of VL cases were also tested for HIV to rule out HIV-VL co-morbidity.

**All VL patients (primary and relapse) were tested for HIV on admission. We have added to the text (Methods) that ‘all’ means ‘all primary and relapse VL patients’. Since implementation of routine HIV screening 98.7% of VL patients had their HIV status confirmed.**

14. Page 6: “Relapse patients were treated either with SSG (20 mg/kg for 30 days) in combination with PM (15 mg/kg for 17 days) or AmBisome.” Dosage of AmBisome?

**We have added the missing dosage information (6 IV infusions of 5 mg/kg on alternate days).**

15. Page 6: “Patients with an increased risk of treatment failure or relapse, i.e. patients with a prior episode of VL, or patients with inadequate or doubtful clinical response, required a negative parasitological test-of-cure by aspirate microscopy to confirm cure of VL.” How long after the treatment, negative parasitological test-of-cure by aspirate microscopy is followed. It could be mentioned here.

**It takes time for parasitaemia to reduce to undetectable level and patients may still be parasitaemic at end of treatment. Test-of-cure is at 21 days for primary VL patients and 30 days for VL relapse patients. We have added this detail to our Methods.**

16. Page 7: “we aggregated patient-level data into 3-year periods” Which three year periods?

**We have clarified that the 3-year periods were 2001-2003, 2013-2015, and 2016-2018.**

17. Table 2: “VL severity score” This could be explained somewhere, what is VL severity score?

**We have added a paragraph to our Methods describing the patient-level clinical variables and VL severity score, as follows: “****A VL severity score was derived from risk factors for VL mortality: age, nutritional status (weight-for-height Z-score for children and adolescents aged <19 years, BMI for adults aged 19+ years), haemoglobin (Hb), and level of weakness. Patients with a risk score associated with >20% probability of death were classified as having ‘severe’ VL and received AmBisome, aggressive antibiotic treatment and nutritional care.*”***

18. Page 25: “The main limitation of our study is that routinely-collected demographic and clinical data from resource-poor and conflict-affected settings will be subject to errors of measurement, recording and classification, although these are more likely to be random than systematic.” Any attempt or method used to minimize this error, can be mentioned here.

**The statistical methods that we used to test for differences in characteristics of primary and relapse VL cases (Chi-squared tests for categorical variables, non-parametric K-sample test, test-for-trend, and Student’s t test for continuous variables) and to estimate odds ratios for VL relapse by HIV and TB status (logistic regression adjusted for age and sex) cannot minimize measurement error, which will tend to bias results towards the null. We have appended this clarification to the end of the quoted sentence.**

19. Page 26: “The high proportion of relapses presenting to our clinic indicate the importance of encouraging patients to return when symptoms re-occur, and of the necessity of close population-level monitoring by national control programs in the region of all VL treatment regimen.” National programme can release a guidance on post treatment follow up protocol for VL treated cases. Incentive to health worker may be included for motivation like Indian setting.

**We have added a sentence here stating that “*However, whether strategies that ensure*** ***6-month or 12-month follow-up can be translated from stable settings such as India or relatively well-resourced effectiveness studies to the South Sudanese context is doubtful at present [30]*”, where ref. #30 is Goyal *et al.* ‘Field effectiveness of new visceral leishmaniasis regimens after 1 year following treatment within public health facilities in Bihar, India’. *PLoS Negl Trop Dis* 2019, 13(9):e0007726.**

20. Page 26: “As miltefosine is teratogenic, precautions will need to be taken when administering it to women of child-bearing age” And pregnant woman.

**We have added “*and to prevent administration during pregnancy*”.**
