# Supplementary material for: The increasing incidence of visceral leishmaniasis relapse in South Sudan: A retrospective analysis of field patient data from 2001–2018
Source: PLoS Negl Trop Dis. 2022 Aug 18;16(8):e0010696. doi: 10.1371/journal.pntd.0010696 (PMC9426874; doi:10.1371/journal.pntd.0010696)
Supplement: Attachment — Submitted filename: Reviewer comments.docx [file pntd.0010696.s002.docx]

The article is well written and can be published with a few changes as below.

I have a few points

1. What is the definition of relapse /Re-infection?

2. How were the cases diagnosed?

3. What are the side effects of the drugs being used for eg SAG , PM,LAMB ?

4. Is 30 mg/kg recommended for LAMB in VL?

5. What were the factors associated with relapse?

6. What was the treatment of HIV-VL and VL-TB?

7. What were drug interactions observed?

8. What was the follow up period?

9. What was the rescue therapy?

10. How many cases converted to PKDL and after what length of time?

11. Is any elimination program currently running in Sudan?
